# Single-nucleotide polymorphisms in *FLT3*, *NLRP5*, and *TGIF1* are associated with litter size in Small-tailed Han sheep

**DOI:** 10.5194/aab-64-475-2021

**Published:** 2021-12-17

**Authors:** Si Chen, Lin Tao, Xiaoyun He, Ran Di, Xiangyu Wang, Mingxing Chu

**Affiliations:** Key Laboratory of Animal Genetics, Breeding and Reproduction, Ministry of Agriculture and Rural Affairs, Institute of Animal Science, Chinese Academy of Agricultural Sciences, Beijing 100193, PR China

## Abstract

Previous studies have indicated that *FLT3*, *NLRP5*, and *TGIF1* play a pivotal role in sheep fecundity. Nevertheless, little is known about the association of the polymorphisms of these genes with litter size (LS). In this study, the selected single-nucleotide polymorphisms (SNPs) were genotyped using a Sequenom MassARRAY® platform, and the distribution of different genotypes of the SNPs in the seven sheep breeds (Small-tailed Han, Hu, Cele Black, Suffolk, Tan, Prairie Tibetan, and Sunite sheep) were analyzed. The reliability of the estimated allele frequency for all seven SNPs was at least 0.9545. Given the association of the *TGIF1* g.37866222C > T polymorphism with LS in Small-tailed Han sheep (p<0.05), fecundity differences might be caused by the change in amino acid from proline (Pro) to serine (Ser), which has an impact on secondary, tertiary protein structures with concomitant *TGIF1* functionality changes. The *FLT3* rs421947730 locus has a great effect on the LS (p<0.05), indicating that the locus of *FLT3* in synergy with *KILTG* is likely to facilitate ovarian follicle maturation and ovulation. Moreover, *NLRP5* rs426897754 is associated with the LS of the second and third parities (p<0.05). We speculate that a synonymous variant of *NLRP5* may be involved in folliculogenesis accompanied by BMP15, FSHR, BMPR1B, AMH, and GDF9, resulting in the different fecundity of Small-tailed Han sheep. Our studies provide valuable genetic markers for sheep breeding.

## 1 Introduction

Many physiological parameters involved in animal reproduction have an important role in the productivity of farm animals. Advances in research on reproduction have enabled the identification of strategies to improve reproductive and productive capacity in livestock. For instance, it has been established that melatonin administration can be used to modify seasonal breeding patterns in goats and sheep (Deveson et al., 1992, Mura et al., 2010) or to decrease milk yield by altering prolactin concentrations in lactating ewes (Molik et al., 2013). Moreover, available evidence suggests that as day length decreases during the autumn, an increase in the duration of melatonin secretion triggers an endocrine response, leading to the onset of reproductive activity (Chemineau et al., 1987). Manipulation of photoperiod and melatonin treatment are widely used for the control of reproduction in goats and sheep (Carcangiu et al., 2015, Giannetto et al., 2020, 2021). Fecundity, one of the significant economic indicators on sheep breeds, has a direct effect on the economic benefit of sheep. The measure of sheep fertility is the litter size (LS) of ewes; thus, increasing the LS plays a pivotal role in developing the sheep industry. The lambing character is quantitative in nature and is controlled by minor polygenes. Molecular markers associated with a quantitative trait can select the genotype directly. That is, combining traditional selection with modern molecular breeding technology greatly boosts the efficiency of sheep breed selection, further improving reproductive performance (Notter, 2012).

During the last decade, microsatellites have been the marker of choice for most parentage and assignment studies because of their high variability and wide availability. Microsatellites are DNA regions composed of a short sequence of two to six nucleotides repeated several times that show high levels of intraspecific allele polymorphism and are widely distributed within genomes of both terrestrial and marine vertebrates (Dekkers, 2014, Arfuso et al., 2017). Furthermore, single-nucleotide polymorphisms (SNPs), which identify the polymorphisms of single nucleotides, are considered useful molecular markers in several research fields, such as LS (Mahdavi et al., 2014, Chong, et al., 2018, Wang et al., 2018).

*FLT3* (Fms Related Receptor Tyrosine Kinase 3) mainly exists in haematopoietic stem cells to regulate hematopoiesis (Lyman and Jacobsen, 1998). Upon stimulation with the FLT3 ligand, the increasing activity of *FLT3* triggers intracellular signaling, such as the PI3K cascade, promoting preimplantation embryo development (Nishijima et al., 2014). In contrast to wild-type *FLT3* signaling, mutated *FLT3* potently triggers the STAT5 pathway (Takahashi, 2011). STAT5 induces cycling D1, antiapoptotic gene p21, and c-myc to be involved in cell growth (Takahashi et al., 2004). These effects may indicate the roles of SNPs of *FLT3* in the reproductive traits, such as LS. A recent study reported that the *FLT3* gene is expressed in oocytes, and its protein is expressed on the oocyte membrane, indicating that *FLT3* modulates follicle growth and maturation similar to CSF-1R and c-kit (Okamura et al., 2001).

*NLRP5* (NLR Family Pyrin Domain Containing 5), as a maternal-effect gene, is essential for embryonic progress and female fertility (Velummailum, 2011). Female mice with *NLRP5* deletion undergo normal process from oogenesis and folliculogenesis to ovulation (Tong et al., 2004). In addition, they normally respond to the stimulation of exogenous gonadotropin, producing a similar number and morphology of oocytes to wild type animals (Tong et al., 2004). Nevertheless, *NLRP5*-deficient mice have meiotic spindle defects in oocytes, which may increase DNA damage (Ohsugi et al., 2008). Therefore, *NLRP5* may be an integral component that functions to preserve the genome integrity of female gametes (Velummailum, 2011).

*TGIF1* (TGFβ Induced Factor Homeobox 1) has been reported to mediate the gonadotropin-releasing hormone (GnRH) pulse sensitivity of FSH-β (Mistry et al., 2011). Under a slow GnRH pulse frequency, overexpressed corepressor *TGIF1* suppress the activation of the FSH-β promoter but has little effect on the fast pulse frequency (Hyman et al., 2003). On the contrary, knockout *TGIF1* selectively increases the expression level of FSH-β mRNA on the fast GnRH pulse frequency (Mistry et al., 2011); that is, *TGIF1* modulates FSH synthesis and secretion in different ways. Moreover, *TGIF1* is highly expressed in the sheep ovary and functions as a transcriptional repressor on the SMAD-induced TGF-β signaling in vertebrates (Jiang et al., 2014, Guca et al., 2018), indicating that *TGIF1* may be involved in reproductive process.

To reveal the relationship of the polymorphism of *FLT3*, *NLRP5*, and *TGIF1* with LS, this study intends to screen out the loci of SNPs in the seven abovementioned native Chinese sheep breeds and genotype them using a Sequenom MassARRAY® platform. By analyzing the distribution of various genotypes on these SNPs in various sheep groups, it is expected to obtain the loci of the SNPs related to reproductive traits and provide valuable genetic markers for sheep genetics and breeding.

## 2 Materials and methods

### 2.1 Animal, sample preparation, and data measurement

All experimental procedure involving sheep were supported by the Science Research Department (in charge of animal welfare issues) of the Institute of Animal Science, Chinese Academy of Agricultural Sciences (IAS-CAAS, Beijing, China). Ethics approval was offered by the animal ethics committee of IAS-CAAS (no. IAS2020-63).

**Table 1:** The number and source location of ewes used in this study.

Breed	Number	Type	District
Small-tailed Han sheep	384	Polytocous and year-round estrus	Shandong Province, China
Hu sheep	83	Polytocous and year-round estrus	Zhejiang Province, China
Cele Black sheep	68	Polytocous and year-round estrus	Cele County, Xinjiang Uygur Autonomous Region, China
Prairie Tibetan sheep	80	Monotocous and seasonal estrus	Danxiong County, Tibet Autonomous Region, China
Sunite sheep	70	Monotocous and seasonal estrus	Inner Mongolia, China
Suffolk sheep	60	Monotocous and seasonal estrus	Beijing, China
Tan sheep	23	Monotocous and seasonal estrus	Ningxia Hui Autonomous Region, China

As shown in Table 1, a total of 768 sheep aged around 3 years were selected, including three higher-fecundity breeds (Small-tailed Han sheep with three consecutive lambing periods, n=384; Hu sheep, n=83; Cele Black sheep, n=68) and four lower-fecundity breeds (Suffolk sheep, n=60; Tan sheep, n=23; Prairie Tibetan sheep, n=80; Sunite sheep, n=70). Blood sampling was performed at the same time. Blood samples were collected into ethylenediaminetetraacetic-acid-coated tubes (Rongxin Bio-Tech Co., Ltd., Beijing, China) via jugular vein catheters (10 mL of blood from each animal) and were stored at -20 ${}^{\circ }C$ for further research. The lambing season, number of births, and litter size were then recorded in turn.

### 2.2 DNA extraction and detection

DNA extraction was carried out as per the blood genomic DNA extraction kit (Tiangen Biochemical Technology Co) instructions. The purity and concentration of DNA were detected using a NanoDrop 2000 spectrophotometer, and the tissue DNA's integrity was tested using 1.5 % agarose gel electrophoresis.

### 2.3 Design of the primer and synthesizing and genotyping of the locus

Combined with our previous data on 768 sheep from seven breeds, which was collected via whole-genome resequencing, some SNPs were screened out (Zhang et al., 2019, 2020; He et al., 2019), including rs421947730 and rs160510212 of *FLT3*; rs429977514, rs408772397, and rs426897754 of *NLRP5*; and g.37866222C > T and rs1092900782 of *TGIF1*. Primers to genotype these SNPs were designed by MassARRAY® Assay Design (v.3.1). MassARRAY® is a strategy for the detection of SNPs, including amplifying target sequences and performing single-base extended polymerase chain reactions (PCRs) with a specific primer (Ellis and Ong, 2017). Due to the fact that various bases occur at polymorphic sites, the amplified products have differences in their molecular weight following extension. These differences enable the products to be separated on the basis of genotype using time-of-flight mass spectrometry. The primers were synthesized by Beijing Tianyi Huiyuan Biotechnology Co. The primer sequences are shown in Table 2.

**Table 2:** Primer information for seven SNPs.

Primer name	Usage	Sequences (5${}^{\prime }$–3${}^{\prime }$)	Product size
FLT3-1-F	PCR for rs421947730	ACGTTGGATGACCAGCCAGGACAGTATATC	115
FLT3-1-R		ACGTTGGATGCCACCCTCTTCACTTACTTC	
FLT3-1-E		CGTCCATGCAGAAAATGATGA	
FLT3-2-F	PCR for rs160510212	ACGTTGGATGCCATCACTATTTCAGGACCA	137
FLT3-2-R		ACGTTGGATGTGAAAATCTCCGTCCACGTC	
FLT3-2-E		TTTTGAATACTGTTGCTACGG	
NLRP5-1-F	PCR for rs429977514	ACGTTGGATGAGTTTGTTCGCTTGGCCTTG	98
NLRP5-1-R		ACGTTGGATGAGGACACCGTTAAGTCCATC	
NLRP5-1-E		TCTGCCATCGGCCGGTTCA	
NLRP5-2-F	PCR for rs408772397	ACGTTGGATGAAGAAGTGTGGCTGCAGTTG	118
NLRP5-2-R		ACGTTGGATGGACATCCAGTCGAACTTTCC	
NLRP5-2-E		TGGACTTAACGGTGTC	
NLRP5-3-F	PCR for rs426897754	ACGTTGGATGTTAACGGTGTCCTCCTTCTG	118
NLRP5-3-R		ACGTTGGATGCATGCCTCAGCAGACTCATC	
NLRP5-3-E		ACTGGATGTCAGAGAGAC	
TGIF1-1-F	PCR for g.37866222C > T	ACGTTGGATGTTCCGCTTCCCTCGAAGCC	98
TGIF1-1-R		ACGTTGGATGCTCCCCGGAGATTCCTCAG	
TGIF1-1-E		TTATTCCTCAGGGACCG	
TGIF1-2-F	PCR for rs1092900782	ACGTTGGATGATGGCCAACAGCAGCTTTAC	100
TGIF1-2-R		ACGTTGGATGTGTGTGTTTGCACTTGGTCC	
TGIF1-2-E		TCCTCTGCGTACATGAG	

### 2.4 Statistical analysis

PopGene32 (version 3.2) software and the PIC-CALC procedure were used to process the results obtained from SNP genotyping and to calculate the PIC (polymorphism information content), HE (heterozygosity), and p values ($\chi^{2}$ test). The particular sheep population, with p>0.05 ($\chi^{2}$ test), was regarded to conform to a Hardy–Weinberg equilibrium (Hui and Burt, 2020). The following animal model was applied: $y_{ijkl}=\mu +{\text{Genotype}}_{i}+P_{j}+I_{k}+e_{ijkl}$. Here, $y_{ijkl}$ is the phenotypic value observed in lambing; μ refers to the average population; ${\text{Genotype}}_{i}$ is the effect of the ith genotypes (i=1,2,3); $P_{j}$ represents the effect of the jth parity (j=1,2,3); $I_{k}$ indicates the parity–genotype interaction effect; and $e_{ijkl}$ is the symbol of random errors, which are supposed to be independent of each other and follow a $N∼(0,\sigma^{2})$ distribution.

The reliability was calculated using the formulas given in the following, ensuring that the estimation of allele frequencies did not deviate from the true value by more than 0.5 times and ensuring the relative deviation when the reliability was at least 0.9545 (Chang et al., 2000).

$$\begin{array}{cc} \beta = & \;\int_{0}^{\lambda }\frac{2e\frac{-\lambda^{2}}{2}}{\sqrt{2\pi }}\;d\lambda ;\\ \eta = & \;2\left[V\left(P^{\frac{1}{2}}\right)\right]\times P^{-1};\\ \lambda = & \;0.5P/{\left[V\left(P\right)\right]}^{\frac{1}{2}};\\ V(P)= & \;\frac{pq}{2n}. \end{array}$$

Here, β, η, λ, P, and V(P) stand for the reliability that allele frequencies do not deviate from the true value more than 0.5 times, the relative deviation when the reliability is at least 0.9545, the standard deviation from the estimate, gene frequencies, and gene variances, respectively.

### 2.5 Predictive protein interaction work of the target genes

Figuring out the protein–protein interactions contributes to deeply understanding the molecular mechanism of *FLT3*, *NLRP5*, and *TGIF1* for complex reproductive traits, such as the LS. The respective transmembrane domains in *FLT3*, *NLRP5*, and *TGIF1* were predicted by TMHMM Server (v.2.0, https://services.healthtech.dtu.dk/service.php?TMHMM-2.0, last access: March 2021). Prediction of the secondary structure, solvent accessibility, ConSurf (Ashkenazy, 2016), and binding site of *TGIF1* were carried out using PredictProtein (https://www.predictprotein.org/, last access: March 2021). The STRING database (v.11.0) was applied to collect and integrate these functional interactions by consolidating protein–protein association data for massive organisms.

## 3 Results

### 3.1 Blood DNA test results

The NanoDrop 2000 and 1.5 % agarose gel electrophoresis were used to detect the purity, concentration, and integrity of tissue DNA from the seven respective breeds. The test results showed ratios of D260 nm/D280 nm between 1.8 and 2.0, all concentrations above 30 $\mathrm{ng}\;µL^{-1}$, and qualified electrophoresis bands; that is, the results could be used for further analysis.

### 3.2 Position mutation analysis of SNPs in the *FLT3*, *NLRP5*, and *TGIF1* genes

**Figure 1:**
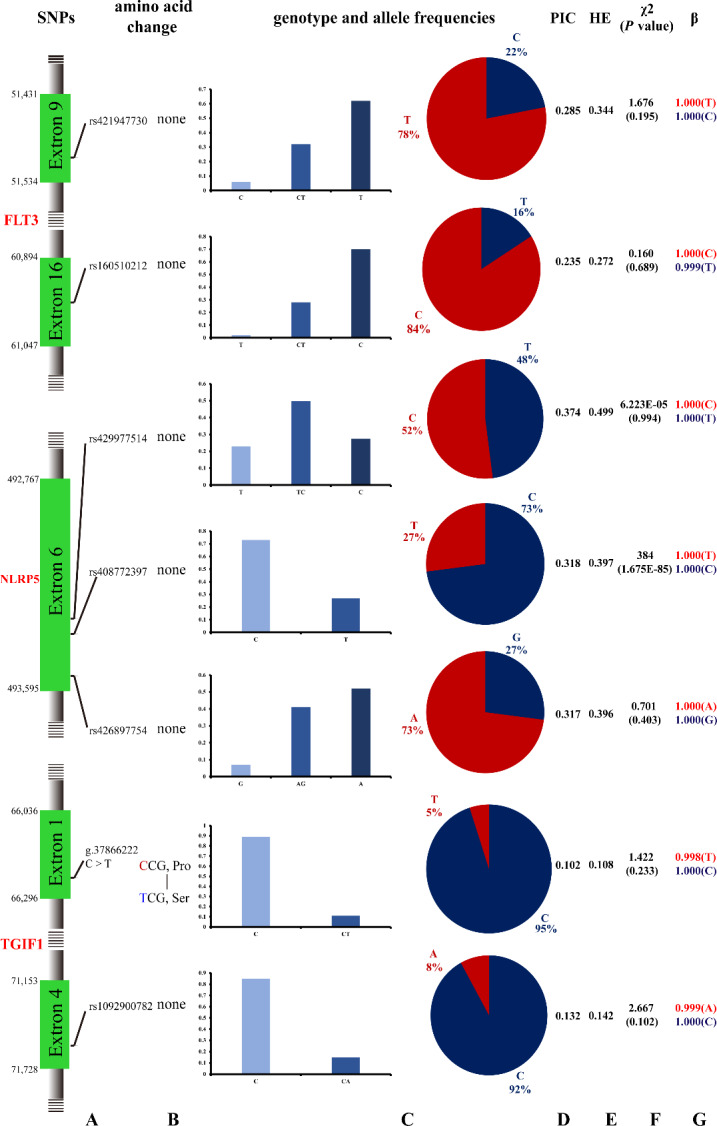
Polymorphisms of the seven SNPs from the *FLT3*, *NLRP5*, and *TGIF1* genes in Small-tailed Han sheep: **(a)** the position of seven SNPs on the chromosome; **(b)** the change in amino acids; **(c)** the genotype and allele frequencies of the *FLT3*, *NLRP5*, and *TGIF1* genes; **(d)** the polymorphism information content (PIC) test; **(e)** the heterozygosity (HE) test; and **(f)** the Hardy–Weinberg equilibrium (HWE) test, $\chi^{2}=\sum (O-E{)}^{2}/E$, where O and E are observed frequency and expected frequency, respectively. **(g)** β represents the reliability of the estimated allele frequencies.

Seven potential SNPs were located in the exon region, including six synonymous variants and one missense variant. Among them, the number of A/G transitions, T/C transitions, and C/A transversions was 1, 5, and 1, respectively, and the phenomenon of T/A, G/C, and G/T transversion was not revealed. The number of transitions accounted for 85.71 % of the total detected SNPs, whereas transversions accounted for 14.29 % (Fig. 1). According to the genotyping results, the *FLT3* gene rs421947730 and rs160510212 possessed two genotypes (CT, TT) in the Tan sheep with low fecundity but three genotypes (CC, CT, TT) in the other six sheep breeds. The *NLRP5* gene rs408772397 possessed two genotypes (CC, TT) in the seven sheep breeds. The other two SNPs of *NLRP5* had three genotypes in all experimental sheep breeds. The *TGIF1* gene g.37866222C > T had two genotypes (CC, CT) in the Small-tailed Han sheep but one genotype (CC) in the other five breeds. The other SNP rs1092900782 had two genotypes (CC, CA) in the seven sheep breeds (Fig. 1, Table 3).

**Table 3:** Population genetic analysis of seven SNPs in six sheep breeds.

Gene	Locus	Breed	Genotype frequency			Allele frequency		PIC	HE	$\chi^{2}$ (p value)
*FLT3*			CC	CT	TT	C	T			
	rs421947730	Hu sheep	0.10	0.29	0.61	0.24	0.76	0.299	0.366	3.644 (0.056)
		Cele Black sheep	0.04	0.38	0.58	0.24	0.76	0.295	0.360	0.266 (0.606)
		Prairie Tibetan sheep	0.04	0.30	0.66	0.19	0.81	0.258	0.305	0.019 (0.891)
		Sunite sheep	0.04	0.30	0.66	0.19	0.81	0.263	0.311	0.093 (0.761)
		Suffolk sheep	0.18	0.45	0.37	0.41	0.59	0.366	0.483	0.283 (0.595)
		Tan sheep		0.17	0.83	0.09	0.91	0.146	0.159	0.209 (0.648)
			TT	CT	CC	T	C			
	rs160510212	Hu sheep	0.05	0.24	0.71	0.17	0.83	0.241	0.280	1.645 (0.200)
		Cele Black sheep	0.05	0.32	0.63	0.21	0.79	0.274	0.327	0.008 (0.930)
		Prairie Tibetan sheep	0.04	0.26	0.70	0.17	0.83	0.241	0.281	0.331 (0.565)
		Sunite sheep	0.01	0.23	0.76	0.13	0.87	0.199	0.224	0.028 (0.867)
		Suffolk sheep	0.02	0.38	0.60	0.21	0.79	0.275	0.330	1.578 (0.209)
		Tan sheep		0.04	0.96	0.02	0.98	0.042	0.043	0.011 (0.915)
*NLRP5*			TT	TC	CC	T	C			
	rs429977514	Hu sheep	0.40	0.44	0.16	0.62	0.38	0.360	0.470	0.362 (0.548)
		Cele Black sheep	0.26	0.50	0.24	0.51	0.49	0.375	0.500	$5.097\times 10^{5}$ (0.994)
		Prairie Tibetan sheep	0.20	0.46	0.34	0.43	0.57	0.370	0.491	0.262 (0.609)
		Sunite sheep	0.23	0.51	0.26	0.49	0.51	0.375	0.500	0.061 (0.807)
		Suffolk sheep	0.35	0.50	0.15	0.60	0.40	0.365	0.480	0.104 (0.747)
		Tan sheep	0.17	0.31	0.52	0.33	0.67	0.343	0.440	2.175 (0.140)
			CC	CT	TT	C	T			
	rs408772397	Hu sheep	0.84		0.16	0.84	0.16	0.229	0.264	83 ($8.205\times 10^{-20}$)
		Cele Black sheep	0.76		0.24	0.76	0.24	0.295	0.360	68 ($1.635\times 10^{-16}$)
		Prairie Tibetan sheep	0.66		0.34	0.66	0.34	0.347	0.447	80 ($3.744\times 10^{-19}$)
		Sunite sheep	0.71		0.29	0.71	0.29	0.325	0.408	70 ($5.930\times 10^{-17}$)
		Suffolk sheep	0.85		0.15	0.85	0.15	0.222	0.255	60 ($9.486\times 10^{-15}$)
		Tan sheep	0.33		0.67	0.33	0.67	0.346	0.444	33 ($9.216\times 10^{-9}$)
			GG	GA	AA	G	A			
	rs426897754	Hu sheep	0.22	0.40	0.38	0.42	0.58	0.369	0.487	2.493 (0.114)
		Cele Black sheep	0.15	0.35	0.50	0.32	0.68	0.438	1.778	2.551 (0.110)
		Prairie Tibetan sheep	0.19	0.45	0.36	0.41	0.59	0.367	0.485	0.410 (0.522)
		Sunite sheep	0.15	0.46	0.39	0.39	0.61	0.362	0.474	0.087 (0.768)
		Suffolk sheep	0.22	0.48	0.30	0.46	0.54	0.373	0.497	0.042 (0.837)
		Tan sheep	0.08	0.22	0.70	0.20	0.80	0.265	0.315	2.200 (0.138)
*TGIF1*			CC	CT	TT	C	T			
	g.37866222C > T	Hu sheep	1.00			1.00	0.00	0.000	0.000	0.000 (0.970)
		Cele Black sheep	1.00			1.00	0.00	0.000	0.000	0.000 (0.970)
		Prairie Tibetan sheep	1.00			1.00	0.00	0.000	0.000	0.000 (0.970)
		Sunite sheep	1.00			1.00	0.00	0.000	0.000	0.000 (0.970)
		Suffolk sheep	1.00			1.00	0.00	0.000	0.000	0.000 (0.970)
		Tan sheep	1.00			1.00	0.00	0.000	0.000	0.000 (0.970)
			CC	CA	AA	C	A			
	rs1092900782	Hu sheep	0.73	0.27		0.87	0.13	0.203	0.230	1.937 (0.164)
		Cele Black sheep	0.97	0.03		0.99	0.01	0.029	0.029	0.015 (0.902)
		Prairie Tibetan sheep	0.96	0.04		0.98	0.02	0.036	0.037	0.029 (0.864)
		Sunite sheep	0.89	0.11		0.94	0.06	0.102	0.108	0.257 (0.612)
		Suffolk sheep	0.98	0.02		0.99	0.01	0.016	0.017	0.004 (0.948)
		Tan sheep	0.91	0.09		0.96	0.04	0.080	0.083	0.048 (0.827)

### 3.3 Population genetic analysis in the six sheep breeds

Besides Small-tailed Han sheep, the population genetic results of seven SNPs in the other six sheep breeds (Hu, Cele Black, Prairie Tibetan, Sunite, Suffolk, and Tan sheep) are shown in Table 3. In the *FLT3* gene, the rs421947730 locus had low polymorphism (PIC≤0.25) in Tan sheep but moderate polymorphism (0.25<PIC<0.5) in the other sheep breeds; it was also under Hardy–Weinberg equilibrium (HWE, p>0.05) in all of the sheep breeds. The rs160510212 locus had moderate polymorphism in Cele Black and Suffolk sheep but low polymorphism in other groups; it also corresponded to the HWE in all groups. In the *NLRP5* gene, both the rs429977514 and rs426897754 loci had moderate polymorphism and were under HWE in the six sheep breeds. The rs408772397 sites had low polymorphism in Hu and Suffolk sheep but moderate polymorphism in the residual sheep breeds; these loci significantly deviated from HWE in all of the sheep breeds (p≤0.05). In the *TGIF1* gene, the g.37866222C > T and rs1092900782 loci had low polymorphism and were under HWE in the six sheep breeds (Table 3). Moreover, the reliability of estimated allele frequencies was at least 0.9545 in all cases.

### 3.4 Polymorphism analysis of the locus in the Small-tailed Han sheep

As is shown in the Fig. 1, the reliability of the estimated allele frequencies was at least 0.954 in all cases. The $\chi^{2}$ test indicated that one SNP (rs408772397) significantly deviated from Hardy–Weinberg equilibrium (p≤0.05). Moreover, the SNP g.37866222C > T, whose base C is replaced by base T, resulting in proline (Pro) substituted for serine (Ser), is described for the first time in this study.

### 3.5 The association of *FLT3*, *NLRP5*, and *TGIF1* polymorphisms with litter size in Small-tailed Han sheep

Litter sizes (LSs) with three parities of different loci were recorded in this study (Table 4). In the first parity, the LS with the mutant type was greater than that for the wild type at most loci. The rs421947730 locus of *FLT3* was associated with the LS at the third parity, and ewes with the CT variant had the highest lambing rate (p<0.05). The rs426897754 locus of *NLRP5* was related to the LS at the second and third parity, and mutant-type sheep with GA had the highest lambing rate (p<0.05). The g.37866222C > T locus of *TGIF1* was associated with the LS at the second and third parity, and mutant-type ewes (CT) were associated with the largest LS (p<0.05); that is, mutant heterozygote ewes produced the maximum LS in our results.

**Table 4:** LSM (least square means)±SE (standard error) of the litter size of three different parities in Small-tailed Han sheep with different genotypes. Note that the different subscript letters in the table indicate differences among the groups (p<0.05).

Locus	Genotype	The first parity LS	The second parity LS	The third parity LS
		(sample size)	(sample size)	(sample size)
rs421947730	CC	1.74±0.14 (23)	2.00±0.22 (12)	$2.08\pm 0.41^{b}$ (4)
	CT	1.91±0.06 (123)	2.31±0.09 (71)	$2.70\pm 0.16^{a}$ (27)
	TT	1.86±0.04 (237)	2.17±0.06 (145)	$2.38\pm 0.11^{b}$ (60)
rs160510212	TT	1.78±0.22 (9)	2.00±0.54 (2)	
	TC	1.85±0.07 (106)	2.03±0.10 (60)	2.39±0.17 (23)
	CC	1.89±0.04 (267)	2.14±0.06 (166)	2.45±0.10 (67)
rs429977514	TT	1.93±0.07 (87)	2.31±0.11 (45)	2.69±0.21 (16)
	TC	1.87±0.05 (191)	2.16±0.07 (122)	2.50±0.12 (48)
	CC	1.82±0.07 (105)	2.11±0.10 (61)	2.32±0.16 (27)
rs408772397	CC	1.89±0.04 (279)	2.08±0.06 (168)	2.42±0.11 (64)
	TT	1.91±0.07 (105)	2.21±0.10 (61)	2.52±0.16 (27)
rs426897754	GG	1.78±0.11 (35)	$1.83\pm 0.18^{b}$ (18)	$1.86\pm 0.42^{b}$ (4)
	GA	1.87±0.05 (157)	$2.06\pm 0.08^{a}$ (98)	$2.32\pm 0.16^{a}$ (28)
	AA	1.90±0.05 (191)	$2.20\pm 0.07^{a}$ (112)	$2.46\pm 0.11^{a}$ (57)
g.37866222C > T	CC	1.86±0.04 (339)	$2.05\pm 0.05^{b}$ (196)	$2.34\pm 0.10^{b}$ (78)
	CT	1.93±0.10 (44)	$2.50\pm 0.13^{a}$ (32)	$2.87\pm 0.23^{a}$ (13)
rs1092900782	CC	1.87±0.04 (324)	2.12±0.05 (196)	2.40±0.10 (77)
	CA	1.88±0.09 (59)	2.16±0.13 (32)	2.61±0.22 (14)

### 3.6 Bioinformatic analysis of *FLT3*, *NLRP5*, and *TGIF1*

**Figure 2:**
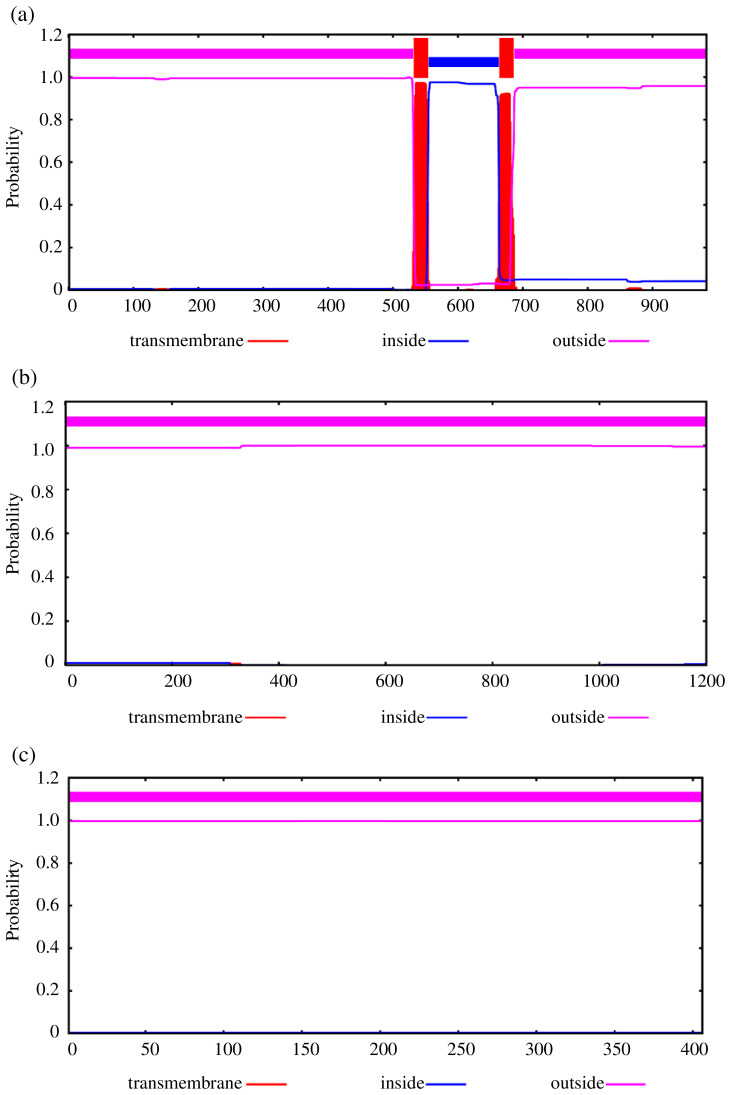
Transmembrane regions' prediction using the amino acid sequence reported in TMHMM, including *FLT3* **(a)**, *NLRP5* **(b)**, and *TGIF1* **(c)**.

**Figure 3:**
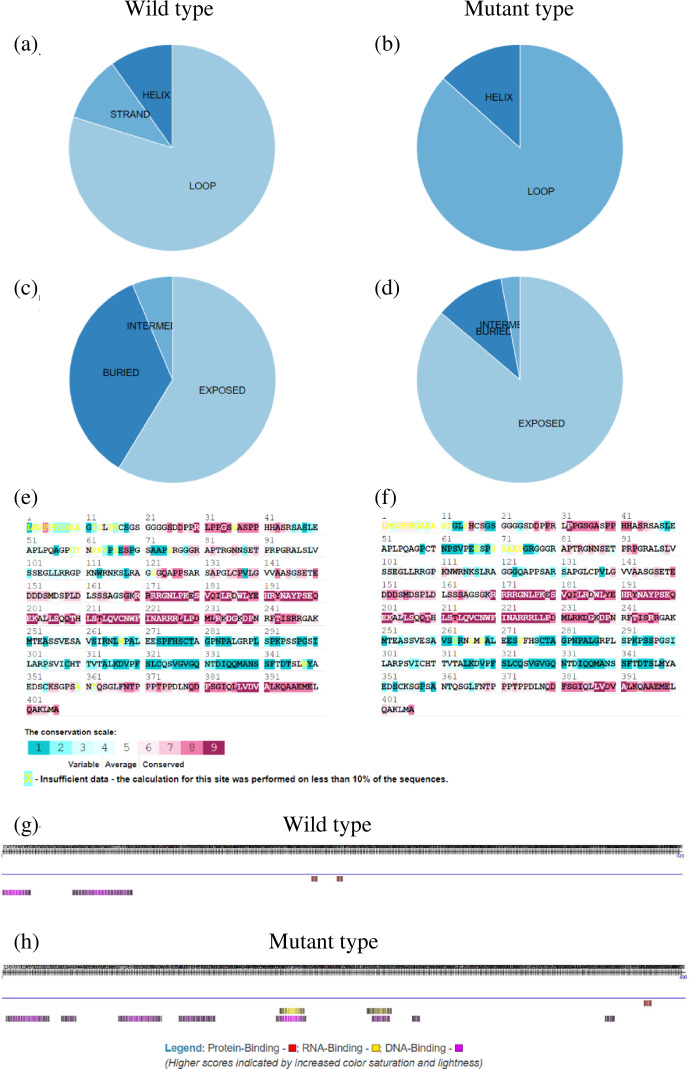
Secondary protein structure for the *TGIF1* product at g.37866222C > T based on its predicted amino acid sequence: **(a, b)** secondary structure composition of wild and mutant type, respectively; **(c, d)** solvent accessibility of wild and mutant type, respectively; **(e, f)** ConSurf results of wild and mutant type, respectively; and **(g, h)** binding site prediction of wild and mutant type, respectively.

**Figure 4:**
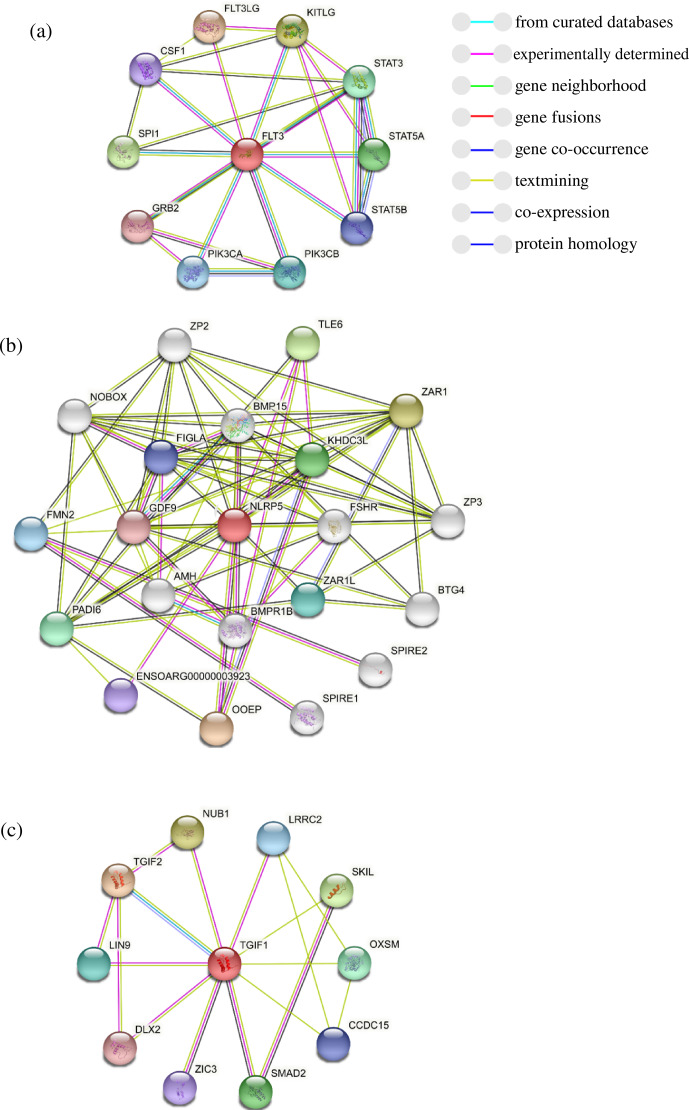
The prediction of the protein interaction networks of *FLT3* **(a)**, *NLRP5* **(b)**, and *TGIF1* **(c)**, based on the STRING database.

To further explore the changes before and after the mutation of genes, some bioinformatic methods were applied, such as transmembrane domain prediction (TMHMM), protein prediction (PredictProtein), and functional protein association networks (STRING). The amino acid residues 1–531 were located outside, 532–554 were located in the transmembrane region, 555–663 were located inside, 664–686 were located in the transmembrane region, and 687–983 were located outside of the FLT3 protein membrane. The synonymous variants of rs421947730 and rs160510212 were set outside of the *FLT3* protein membrane and the transmembrane regions, respectively. Moreover, the amino acid residues 1–1200 and 1–406 were located outside of the *NLRP5* and *TGIF1* protein membrane, respectively (Fig. 2). That is, the replacement of Pro with Ser at position 63 of g.37866222C > T occurred outside of the *TGIF1* protein membrane. The analysis of protein prediction indicated that the obvious differences in *TGIF1* g.37866222C > T existed in the secondary structure, solvent accessibility, ConSurf, and binding site prediction (Fig. 3). This missense variant induced a change in the surrounding sites from average or conserved to variable and also increased the number of protein-binding sites, RNA-binding sites, and DNA-binding sites, indicating that g.37866222C > T might affect *TGIF1* function with respect to reproduction. Protein interaction networks indicated that *FLT3*, *NLRP5*, and *TGIF1* were predicted to interact with the known proteins affecting the ovulation rate and LS, such as *FLT3* with KITLG; *NLRP5* with BMP15, GDF9, AMH, BMPR1B, and FSHR; and *TGIF1* with SMAD2 (Fig. 4).

## 4 Discussion

Marker-assisted selection with traditional selection is potentially effective for the improvement of traits that are of low heritability, such as ovulation rate and LS (Williams, 2005). *FLT3* and *NLRP5* are both expressed in oocytes and are involved in follicle growth and maturation to increase the ovulation rate (Okamura et al., 2001, Bebbere et al., 2014, Velummailu, 2011). *TGIF1*, a SMAD transcriptional corepressor, negatively dictates the TGF-β signaling pathway to repress reproduction (Guca et al., 2018). The rs421947730 and rs160510212 loci of *FLT3*, rs408772397 of *NLRP5*, and g.37866222C > T and rs1092900782 of *TGIF1* exhibited low polymorphism (PIC≤0.25), which may be limited by the sample size. Due to selection, several sheep breeds were not in HWE (p≤0.05) at these loci. This study reveals that some SNPs of these genes are associated with the LS in Small-tailed Han sheep.

Missense mutations have a great effect on complex traits. Point mutations in *GDF9* (Tong, 2020), *BMPR1B* (Souza, 2001), and *BMP15* (Hanrahan, 2004) have been reported to significantly affect the LS in sheep. Similar to previous studies, our study revealed that the *TGIF1* missense variant g.37866222C > T showed a significant increase in LS at the second and third parity and that mutant-type ewes (CT) displayed the maximum LS (p<0.05). However, the mutant homozygous genotype TT was not detected, similar to g.37871539C > T in *TGIF1* (Zhang, 2020), indicating that lethality may be caused by mutant homozygous genotypes at some loci of *TGIF1* in all sheep breeds. Moreover, cellular transmembrane domains enable numerous proteins to function on the fertility-related signaling pathway (Guna and Hegde, 2018). In our results, the replacement of Pro with Ser at g.37866222C > T occurred outside of the *TGIF1* protein membrane, indicating that g.37866222C > T might be a ligand-binding site. Given that *TGIF1* interacts with *SMAD2* via protein–protein networks, the missense variant decreases critical protein-binding sites, thereby weakening the formation of the protein complex. Once the protein complex has been reduced, the *TGIF1*–SMAD system would not be released from promoters or enhancers, resulting in *TGIF1* being unable to function on TGF-β signaling as an active repressor of SMAD complexes (Guca et al., 2018). Considering the association of g.37866222C >  T polymorphism with LS in Small-tailed Han sheep, fecundity differences might be caused by the change in amino acid from Pro to Ser, which has an impact on secondary and tertiary protein structures, causing *TGIF1* functionality changes.

The *FLT3* gene has been demonstrated to distribute on oocytes, and its protein distributes on the membrane of oocytes, indicating that *FLT3* is involved in various stages of folliculogenesis like c-kit and CSF-1R (Okamura et al., 2001). In the protein–protein networks, *FLT3* is predicted to interact with *KITLG*. In the ovine follicles, *KITLG* mRNA is highly expressed in early antral follicles but reduced at the late antral stage with follicular growth progress (Joyce et al., 2000). *KITLG*–*KIT* interactions exist in several stages of follicular development (Yoshida et al., 1997), and they function on ovarian follicle maturation (An et al., 2015). Thus, *KITLG* may be a candidate gene for the selection of sheep reproductive traits. The rs421947730 locus has a significant difference in the litter size of mutant type (CT, TT) versus wild type (CC), indicating that the locus of *FLT3* in synergy with *KITLG* is likely to facilitate ovarian follicle maturation and ovulation in Small-tailed Han sheep. *NLRP5* wild- and mutant-type females have similar follicular recruitment rates and a similar ovarian germ pool. Nevertheless, ovulated oocytes with *NLRP5* deletion induce premature activation of the mitochondria, followed by embryo demise due to mitochondrial depletion (Fernandes et al., 2012). In the analysis of protein prediction, NLRP5 protein interacts with the proteins related to follicular development, such as BMP15, FSHR, BMPR1B, AMH, and GDF9 (Chu et al., 2007; Feary, et al., 2007). Moreover, at the rs426897754 locus of *NLRP5*, the LS of mutant-type (GA, AA) sheep is higher than that for wild-type (GG) sheep at the second and third parity. We suggest that *NLRP5* with a synonymous variant may be involved in folliculogenesis accompanied by BMP15, FSHR, BMPR1B, AMH, and GDF9, resulting in the different fecundity of Small-tailed Han sheep.

## 5 Conclusions

Among these SNPs, g.37866222C > T of *TGIF1* at the second and third parity, rs421947730 of *FLT3* at the third parity, and rs426897754 of *NLRP5* at the second and third parity can significantly increase the lambing rate of Small-tailed Han sheep (p<0.05). Moreover, bioinformatic analysis indicated changes in secondary and tertiary protein structures after g.37866222C > T mutation in *TGIF1*. With respect to the molecular mechanisms, further verification is required that *FLT3* rs421947730 and *NLRP5* rs426897754 interacting with important proteins could increase the lambing rate. Our study suggests that *TGIF1*, *FLT3*, and *NLRP5* might serve as valuable genetic markers affecting the LS in sheep.

## Data Availability

Data availability is not applicable to this article, as no new data were created or analyzed in this study.
